# Supplementary figures and images for: Archaea Microbiome Dysregulated Genes and Pathways as Molecular Targets for Lung Adenocarcinoma and Squamous Cell Carcinoma
Source: Int J Mol Sci. 2022 Sep 30;23(19):11566. doi: 10.3390/ijms231911566 (PMC9570029; doi:10.3390/ijms231911566)

LUAD

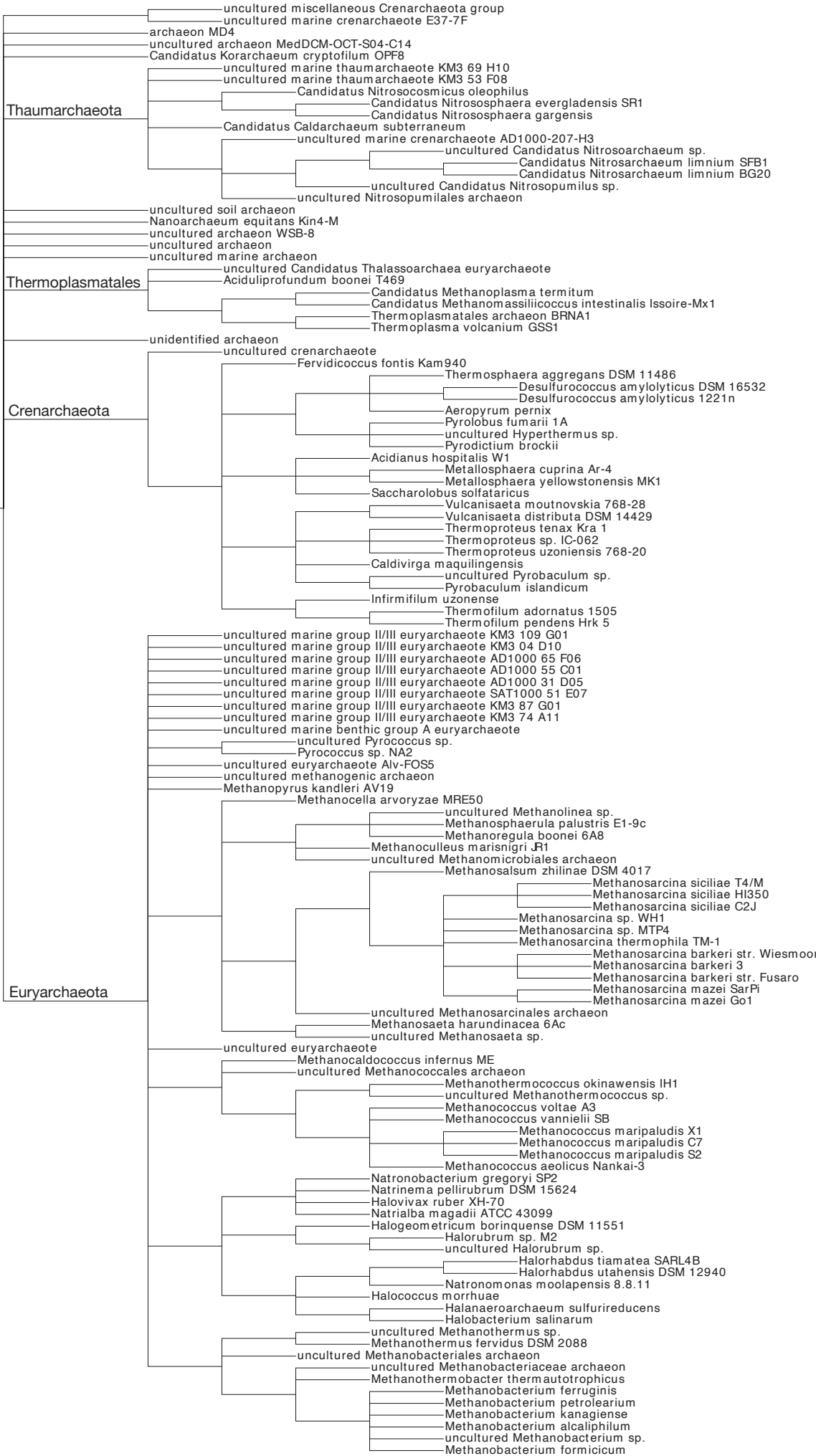

LUSC

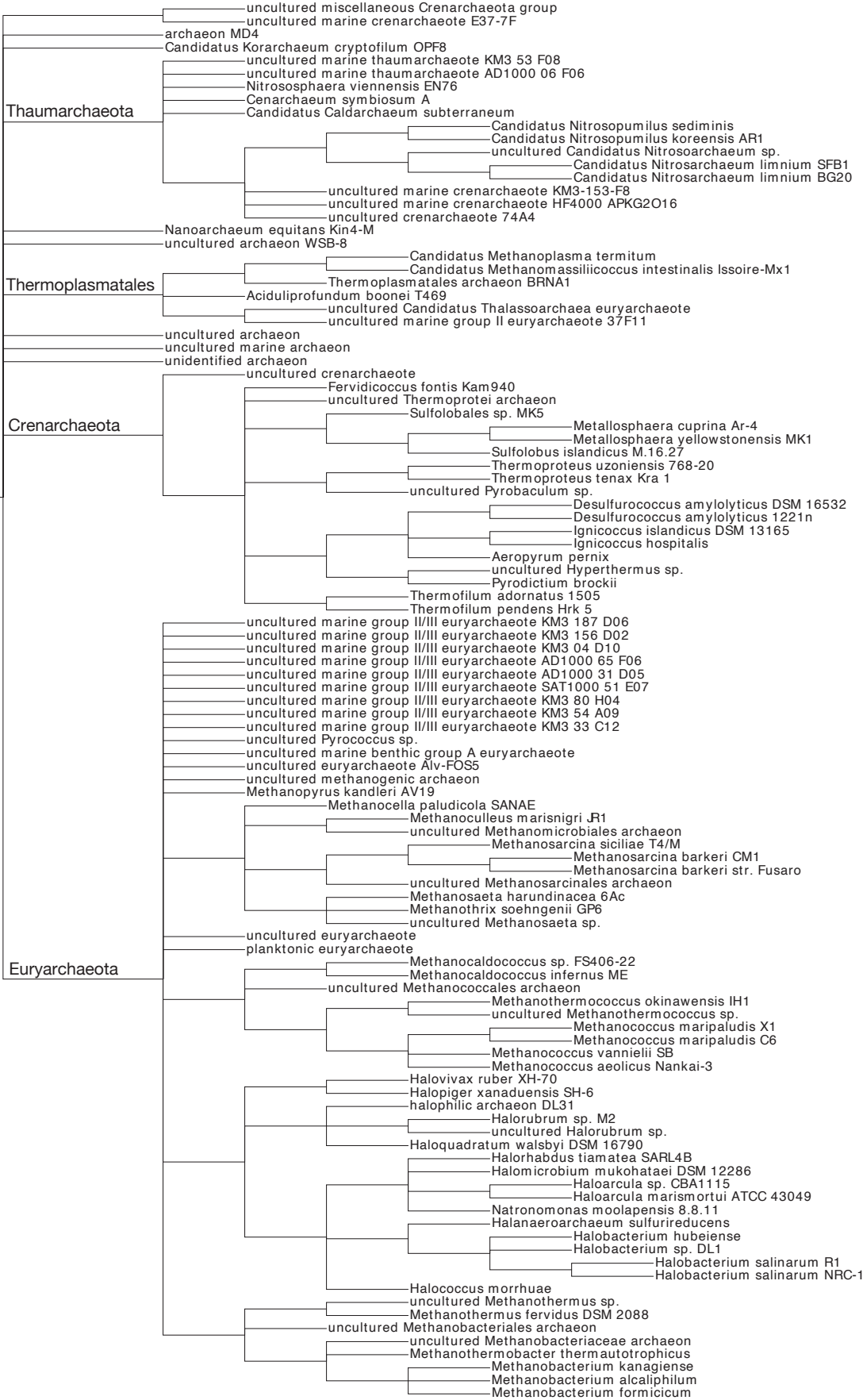

Supplement: Supplementary file 1 [file ijms-23-11566-s001.zip › ijms-1926575-supplementary.pdf]
